# Diagnosis of Pulmonary Embolism: A Review of Evidence-Based Approaches

**DOI:** 10.3390/jcm13133722

**Published:** 2024-06-26

**Authors:** Sneha E. Thomas, Ido Weinberg, Robert M. Schainfeld, Kenneth Rosenfield, Gaurav M. Parmar

**Affiliations:** Vascular Medicine Section, Massachusetts General Hospital, Boston, MA 02114, USA; sthomas013s@gmail.com (S.E.T.); iweinberg@mgh.harvard.edu (I.W.); krosenfield1@mgh.harvard.edu (K.R.)

**Keywords:** pulmonary embolism, venous thromboembolism, deep-vein thrombosis, diagnosis, clinical judgment, blood clots, risk stratification, biomarkers, pulmonary embolism response team, vascular medicine

## Abstract

Venous thromboembolism, commonly presented as pulmonary embolism and deep-vein thrombosis, is a paramount and potentially fatal condition with variable clinical presentation. Diagnosis is key to providing appropriate treatment in a safe and timely fashion. Clinical judgment and assessment using clinical scoring systems should guide diagnostic testing, including laboratory and imaging modalities, for optimal results and to avoid unnecessary testing.

## 1. Introduction

Venous thromboembolism (VTE) is a substantial contributor to the burden of non-communicable diseases globally [1]. VTE includes blood clots formed in the venous circulation, deep-vein thrombosis (DVT), along with blood clots that break off and travel to the pulmonary vasculature, leading to pulmonary embolism (PE) [2]. Despite efforts at prevention and prophylactic measures, the incidence of VTE has been rising in the last several decades [3,4]. Studies report annual incidence rates for PE ranging from 39 to 115 per 100,000 people, while the incidence for DVT ranges from 53 to 162 per 100,000 people [5,6,7]. However, the actual incidence rates are likely significantly higher, as many patients are asymptomatic and many others are underdiagnosed or misdiagnosed. For instance, there are reports of silent PE in 40–50% of patients with proximal DVT and numerous PEs incidentally found upon autopsy [8,9]. Massive PE can lead to elevated physiologic dead space secondary to occlusion in pulmonary vascular flow [10]. Regardless, PE remains a fatal condition with high mortality rates. An epidemiological model created among six countries in the European Union reported an estimated annual total of 465,715 symptomatic DVT cases, 295,982 symptomatic PE cases, and 370,012 VTE-related fatalities [11,12]. Of these deaths, 27,473 (~7%) of the VTE events were identified antemortem, whereas 126,145 (~35%) were deadly PE, and 217,394 (~ 60%) were undiagnosed PE [13]. 

The ICOPER study evaluated 2454 patients with acute PE from seven countries in Europe and North America and showed an overall crude mortality rate of 17.4% at 3 months [2]. PE was attributed as the cause of ~45% of deaths, and ~75% of the fatalities transpired during the initial hospitalization for PE [2]. This highlights the importance of timely diagnosis to initiate treatment promptly and reduce the risk of mortality and morbidity. In this article, we hope to summarize a comprehensive approach to the diagnostic evaluation of PE while also avoiding unnecessary testing in appropriate clinical settings.

## 2. Pretest Probability

### 2.1. Does Clinical Presentation and Physical Examination Have Any Role in PE Diagnosis in 2024?

Clinical manifestations of acute pulmonary embolism can be very nonspecific, given its various presentations (Table 1), and they often result in a differential diagnosis for numerous typical and atypical presentations. The range of presentation varies from no symptoms to mild–moderate symptoms of shortness of breath to severe cases with hemodynamic collapse [14,15]. PE is typically suspected in patients who present with symptoms such as dyspnea, pleuritic chest discomfort, cough, and hemoptysis, with an incidence of 73%, 66%, 37%, and 13%, respectively, based on PE data from the Prospective Investigation of Pulmonary Embolism Diagnosis (PIOPED) study [14,15,16,17]. Syncope is an extensively discussed clinical symptom in PE, given the variable reports of its incidence and prognostic role [16,18,19]. The proposed mechanism for syncope in PE involves pulmonary vascular obstruction by a large embolus, resulting in impaired right ventricular function and consequently impacting left ventricular filling and cardiac output. Hemodynamic instability and circulatory compromise suggestive of right ventricular strain is an infrequent yet significant clinical finding since they may suggest central or widespread pulmonary embolism with a diminished ability to maintain a stable blood flow [16]. Cardiac arrhythmias, the Bezold–Jarisch reflex, hypoxemia, orthostatic dysfunction, and other comorbidities can also precede syncope events [20]. Even though syncope alone may not have a prognostic role, studies have shown elevated risk for early PE-related adverse outcomes such as early mortality (during hospitalization or <30 days) and 30-day negative events in patients with syncope [18]. Common physical examination findings of PE include tachycardia, tachypnea, or pulmonary hypertension/right heart strain, such as jugular venous distension, a loud P2 (pulmonic) component of the second heart, and right ventricular parasternal lift [21]. However, none of these findings are specific enough to diagnose PE, nor does the lack of these findings exclude PE. The presence of clinical symptoms and provoking risk factors for PE enables patients to be classified into distinct pretest probability categories.

### 2.2. Role of Clinical Scoring Systems

Pretest probability assessment may be based on clinical judgment alone or clinical prediction scores. The major disadvantage of depending on clinical judgment alone is the subjectivity in the assessment and the lack of standardization. The clinical scoring systems provide objective variables that can allow for a more standardized approach to assess clinical probability and ultimately lead to a more effective diagnostic process. As a result, the clinical prediction rules may become helpful in assessing pretest probability in scenarios where clinical judgment alone is equivocal. The most commonly used and validated systems to assess pretest probability include the simplified and modified Wells Scoring System and the Revised Geneva Scoring System (Table 2) [22,23].

### 2.3. Clinical Judgement versus Decision Rules

Studies have shown a trend toward increasing accuracy with increasing clinical experience, and there is some uncertainty in the accuracy of the clinical gestalt of inexperienced physicians [24,25,26]. The gestalt of experienced clinicians has demonstrated comparable accuracy in identifying patients with low, moderate, and high pretest probabilities of PE in a few studies [24,25,27]. A retrospective analysis of a prospective cohort of 1038 patients was performed to assess the accuracy of gestalt evaluation compared to the revised Geneva score and the modified Wells score [27]. The area under the curve varied substantially across the three methods. Specifically, the AUC was 0.81 (95% confidence interval (CI) 0.78 to 0.84) for gestalt evaluation while it was 0.71 (95% CI 0.68–0.75) for the Wells score and 0.66 (95% CI, 0.63–0.70) for the revised Geneva score. The study results showed a favorable comparison between gestalt evaluation and clinical decision rules in assessing the clinical probability of PE and especially did better in selecting low- and high-clinical-probability patients [27]. Another meta-analysis of 52 studies involving 55,268 patients compared these different clinical prediction methods and showed comparable results. In 15 studies, gestalt assessment was utilized and showed a sensitivity of 0.85 with a specificity of 0.51. Nineteen studies used Wells scoring with a cutoff < 2, resulting in a sensitivity of 0.84 with a specificity 0.58, while eleven studies used Wells scoring with a cutoff of 4 or less and showed a sensitivity of 0.60 and a specificity of 0.80. Five studies applied the Geneva score and showed a sensitivity of 0.84 and a specificity of 0.50, and finally, four studies adopted the revised Geneva score and showed a sensitivity of 0.91 and a specificity of 0.37 [28]. In summary, it is acceptable to utilize clinical gestalt evaluation (especially by an experienced clinician) or any available scoring systems (Wells or Geneva) to assess the pretest probability of a pulmonary embolism.

## 3. Ruling out PE

### 3.1. PERC Rule

Although identifying patients with PE is critical, it is equally important to avoid the overuse of diagnostic tests for PE and lower inappropriate costs and complications of unnecessary testing, especially when clinical suspicion is low. The Pulmonary Embolism Rule-out Criteria (PERC) rule should be utilized for patients who are considered to have a low probability of PE. The PERC includes the following criteria: individuals 50 years of age or older, heart rate of 100 bpm or higher, oxygen saturation level of <95% while on room air, asymmetric lower-extremity swelling, presence of hemoptysis, recent major surgery or traumatic event, history of prior PE or DVT, and use of any type of exogenous hormones [29,30]. If any of the eight criteria are positive, the PERC rule cannot be used to rule out PE, but if all are negative, the risk of testing is greater than the risk for embolism, and PE can be ruled out with no further testing [30,31,32].

### 3.2. D-Dimer Testing

#### 3.2.1. Different Techniques of D-Dimer Measurement

D-dimer is a soluble fibrin degradation byproduct of coagulation and fibrinolysis. D-dimer has high sensitivity along with high negative predictive value, though it lacks specificity [33,34,35]. D-dimer can help to exclude PE in low and intermediate-risk patients. More specifically, the enzyme-linked immunofluorescence assay (ELFA), microplate enzyme-linked immunosorbent assay (ELISA), and latex quantitative assay have lower specificity but higher sensitivity when compared to other D-dimer assays such as whole-blood D-dimer assay, latex semiquantitative assay, and latex qualitative assay (Table 3A) [34,36]. Consequently, it is imperative to note that some individuals with suspected PE may have negative results [37]. In patients with an intermediate pretest probability of PE, low-risk individuals where the PERC rule cannot be applied, or those with a low pretest probability but do not meet all the criteria to rule out PE, it is recommended to obtain a high-sensitivity D-dimer test as the preliminary diagnostic evaluation [38]. In high-risk patients and for patients with elevated D-dimer, advanced imaging, including computed tomography pulmonary angiography (CTPA), should be considered.

#### 3.2.2. Age Adjustment of D-Dimer

The performance of the D-dimer test is significantly influenced by age [49]. In patients who are 50 years of age or older, the age-adjusted D-dimer threshold (age × 10 ng/mL) should be utilized to assess whether imaging is necessary, instead of a generic threshold of 500 ng/mL [50]. Studies have noted that the pretest clinical probability assessment and age-adjusted D-dimer cutoff together yield a greater proportion of individuals in whom PE can be confidently excluded, with a low likelihood of future VTE events, when compared to a set 500 µg/L cutoff [50]. The YEARS diagnostic algorithm looked at D-dimer cutoff adaptations based on clinical probability (three items from the Wells score and D-dimer level). PE was excluded in patients without clinical items (DVT symptoms, hemoptysis, and alternate diagnosis less likely) and D-dimer < 1000 ng/mL vs. one or more clinical items and D-dimer < 500 ng/mL. A study showed a significant reduction of 14% in computed tomography pulmonary angiography (CTPA) tests across all age groups and other relevant subgroups [51].

Certain special populations other than the elderly also need D-dimer modifications. For example, the ELISA D-dimer test appears to be a reliable way to exclude the presence of PE in cancer patients. However, it yields negative results at the standard cutoff value in only 10% of patients. Elevating the threshold of the cutoff value in cancer patients may enhance the use of the test in this particular population; a negative test can still reliably exclude PE diagnosis, nonetheless [52,53,54]. Similarly, a large proportion of outpatients with suspected pulmonary embolism can be efficiently ruled out by combining D-dimer results with clinical and pretest assessments. For instance, a Wells score of four or lower in conjunction with a negative D-dimer measurement may effectively and safely rule out PE in outpatient settings [55].

D-dimer measurement appears less useful in hospitalized and critically ill patients due to its lower specificity within these specific groups [56]. As such, it is crucial to use clinical judgement and pretest probability in assessing this population. It also remains as a diagnostic challenge in patients with underlying pulmonary etiology such as consolidation or pneumonia [57].

## 4. Role of Other Laboratory Biomarkers

### 4.1. Arterial Blood Gas (ABG)

Although few studies in the past have suggested normal (A–a) O_2_ gradient as a possible PE exclusion criterion, studies in recent years have revoked these findings [58,59,60]. Arterial blood gas (ABG) collected from a study of 293 patients showed that ABG, either by itself or in conjunction with other clinical parameters, has little diagnostic utility when PE is suspected [60]. Similarly, PE could not be ruled out in over 30% of cases in patients without preexisting cardiopulmonary disease when the partial pressure of arterial oxygen was 80 mm Hg or higher, the partial pressure of arterial carbon dioxide was 35 mm Hg or higher, and the P(A–a) O_2_ gradient was 20 mm Hg or lower [61]. Likewise, PE could not be dismissed as a possibility in over 14% of cases under the same conditions in patients with preexisting cardiopulmonary disease [61]. As a result, blood gas levels do not provide enough information to definitively rule out the possibility of PE.

### 4.2. Brain Natriuretic Peptide (BNP)

BNP and N-terminal pro BNP (NT-proBNP), its precursor, are neurohormones produced when the myocardium is stretched [62,63]. PE can lead to stretching of the right ventricle from pressure overload. Although both of these biomarkers are not often beneficial for the diagnosis of PE itself, they are frequently useful for prognostication as they can be considered indirect markers of right ventricular dilation and strain (Table 3B) [64,65,66]. A meta-analysis that reviewed 12 studies noted that higher levels of BNP corresponded with elevated all-cause mortality in the short term (odds ratio, 6.5; 95% CI, 3.1–13.9), mortality-related PE (OR, 6.1; 95% CI, 2.5–14.3), and major adverse events (OR, 7.5; 95% CI, 4.2–13.2) [39,67]. This study has also highlighted that normal BNP is a strong negative predictor in acute PE (Table 3A) [39,43]. However, utility is questionable in patients who may have other etiologies for the elevated BNP/Pro-BNP.

### 4.3. Troponin

Similar to BNP, troponin is also a great prognostic indicator but has minimal diagnostic value. It is a nonspecific marker of myocardial inflammation or injury. Elevated serum troponin suggests poorer immediate and long-lasting effects in individuals with PE (Table 3C) [40]. It may be utilized as an early and reliable marker of right ventricular dysfunction, especially when an echocardiogram is not immediately available [40,68].

### 4.4. Lactate

Serum lactate is a marker of tissue hypoxia. Several clinical conditions affecting perfusion and/or oxygen demand and supply, such as sepsis, may affect the serum lactate concentration. It is also a significant prognostic marker in acute pulmonary embolism. In a study of 270 patients, patients with lactate levels (> or =2 mmol/L) showed a mortality rate of 17.3%, (95% CI, 12–20%), while patients with lower lactate levels had a mortality rate of 1.6% (95% CI, 0.8–2%). Serum lactate level had a significant impact on both the overall mortality and composite endpoints in this study. The hazard ratio for overall mortality was 11.7 (95% CI, 3.3–41.0), while the hazard ratio for the composite endpoint was 8.1 (95% CI, 3.8–17.3). These effects were seen irrespective of the occurrence of shock, hypotension, right ventricular failure, or elevated troponin [69]. Another similar study involving 496 normotensive outpatient participants with acute symptomatic PE and an elevated plasma lactate showed that individuals with higher lactate levels had a higher likelihood of PE-related sequelae with an adjusted odds ratio 5.3 (95% CI, 1.9–14.4; *p* = 0.001) in contrast to those with lower lactate levels [69,70]. The positive predictive value of the combination of high plasma lactate with indices of right ventricular dysfunction on echo and myocardial injury such as cardiac troponin was ~18% (95% CI, 6.1–36.9%), making it an exceptionally beneficial prognostic indicator to assess complications associated with PE in <7 days [70].

## 5. Pulmonary Embolism Severity Index (PESI)

The pulmonary embolism severity index is a prognostic guide that enables the classification of patients with PE into different risk groups based on mortality (Table 4). The PESI rule applies clinical criteria for estimating outcomes within a 30-day period.

## 6. Role of PERT (Pulmonary Embolism Response Team)

Currently, there are no guidelines on the timeframe for diagnosis and management, although clinicians understand the critical nature of the diagnosis, especially in hemodynamically unstable patients. Since Massachusetts General Hospital implemented the first pulmonary embolism response team (PERT) in 2012, several other centers have adopted this multidisciplinary initiative to facilitate the diagnosis and treatment of patient with intermediate–high- and high-risk PE over the past decade [71,72,73]. Although several different structures exist within various small and large institutions, PERT programs generally aim to incorporate team-based multidisciplinary care into PE care by coordinating anticoagulation plans, thrombolytics vs. catheter-directed treatments, surgical options, and follow-ups [74]. The impact of PERT in facilitating multidisciplinary care is crucial, and more data are needed in this area to assess the effect on PE mortality and morbidity [75].

## 7. Role of EKG/ECG

A 12-lead electrocardiogram (EKG) may provide insights on the PE severity, if any acute changes are present. The six EKG findings including heart rate > 100 bpm (38%), S1Q3T3 (24%), complete right bundle branch block (10%), T-wave inversions in leads V1–V4 (29%), ST segment elevation in aVR (36%), and atrial fibrillation (15%) were found to be predictive of circulatory collapse and 30-day mortality following sudden PE in a systematic review and meta-analysis of 3007 patients [76]. However, these EKG changes alone are not enough to make the diagnosis, and a lack of EKG changes does not reliably reject the possibility of PE diagnosis.

## 8. Role of Various Imaging Modalities

### 8.1. Chest X-ray (CXR)

Acute pulmonary embolism is most commonly accompanied by the presence of cardiomegaly on chest radiographs. However, chest radiographs are not helpful in diagnosing pulmonary embolism but rather help exclude other mimickers of PE [77]. Normal CXR is also necessary for accurate and reliable interpretation of the ventilation–perfusion scan.

### 8.2. CT Pulmonary Angiography vs. Lung Scintigraphy

For patients with suspicion for PE, Multidetector Computed Tomographic Pulmonary Angiography, or CTPA, is the preferred imaging modality [78]. A filling defect that appears following contrast administration in any branch of the pulmonary artery is indicative of PE. The PIOPED II study reported a sensitivity of 83% (95% CI, 76–92%) and a specificity of 96% (95% CI, 93–97%) among 773 patients who had CTPA for the diagnosis of PE [78]. CTA-CTV (CT venogram) was also evaluated in the study and showed a sensitivity of 90% and specificity of 95%. Both CTPA and CTA-CTV had high concordance with clinical assessment. However, if there is discordance between the clinical judgment and the CTPA results, further evaluation should be considered. Motion artifacts, large body habitus, artifacts due to foreign objects, and inadequate contrast enhancement of the pulmonary vasculature can all lead to poor study quality [79]. CTV of the pelvis and lower extremity is not routinely performed in all patients unless there are clinical signs, given the risk of radiation, even though it might improve the diagnostic yield [80].

The detection of smaller emboli has increased with the use of newer scanners with higher resolution. For example, segmental and subsegmental artery visualization and interobserver agreement in the detection of PEs have been substantially enhanced by multi-detector row CT. However, the clinical significance of these smaller embolisms is still unclear [81,82].

For several years, lung scintigraphy/ventilation–perfusion (V-Q) scanning used to be the choice noninvasive imaging for patients with suspected PE. Many have had nondiagnostic evaluations due to the inconclusive results, however. CTPA emerged as the primary imaging technique for suspected pulmonary embolism (PE), effectively replacing V/Q scanning in the United States by 2001 [83]. However, V-Q still has utility in specific situations such as severe contrast allergy, severe renal dysfunction, and low radiation risk in pregnant and even young female patients where scintigraphy provides distinct advantages [84]. A normal chest radiograph is necessary given the risk of false positives due to underlying lung pathologies.

The updated PIOPED criterion, with an area under the ROC curve of 0.753, demonstrated greater accuracy compared to the previous PIOPED criteria [17,85]. However, intermediate probability or indeterminate studies remain a major limitation of V-Q scanning.

In another randomized study involving 1417 patients, the V-Q scan was noted to be non-inferior to CTPA in ruling out PE when used in combination with clinical probability evaluation, D-dimer, and lower-extremity ultrasonography [86]. However, it is important to note that despite achieving statistical significance, the V-Q scan group missed one fatal PE, and CTPA detected more patients with PE [86]. For patients at increased risk of pulmonary embolism (PE), employing a diagnostic approach involving chest X-ray and V-Q scanning based on the PISAPED criteria appears to be less safe compared to using CTPA [87].

### 8.3. Role of Magnetic Resonance Angiography

Magnetic resonance angiography (MRA) has not yet become a substitute for CTPA in assessing acute PE. However, it has the potential for specific utility in patients who cannot tolerate iodinated contrast and in pregnant or young patients, similar to a V/Q scan. The current MRI technology has a notable level of accuracy and precision in detecting proximal pulmonary embolism (PE), but its ability to detect distal PE is still limited, resulting in a sensitivity shortfall. Additionally, approximately 30% of the results obtained from this technology are inconclusive. While MRI/MRA can be helpful in clinical decision-making, it cannot be relied upon as the sole diagnostic study to rule out PE [88]. For patients with technically satisfactory images, the combination of magnetic resonance pulmonary angiography and magnetic resonance venography demonstrates a higher degree of sensitivity than magnetic resonance pulmonary angiography alone [89]. Regardless, acquiring technically satisfactory images using both methods is more challenging [90].

### 8.4. Imaging Modalities of the Future

V-Q single-photon emission CT (SPECT) has been reported to provide highly accurate negative and positive predictive values, with just 1% of the results being inconclusive [91]. However, accessibility for this study remains a major limitation, along with varied diagnostic criteria.

V-Q SPECT and low-dose CT without contrast combination has also shown outstanding diagnostic accuracy in a few studies and should be further explored [92]. The utilization of SPECT/low-dose CT can also help distinguish between lung symptoms, leading to a notable enhancement in diagnosing pulmonary embolism or identifying other lung disorders in a substantial number of patients, particularly when anomalies in lung perfusion are observed [93].

### 8.5. Is Pulmonary Angiography Still a Gold Standard?

Pulmonary angiography is the most accurate examination for detecting embolism and used to be the “gold standard”. However, the advancements in noninvasive imaging modalities have changed the criteria for doing angiography. It is rarely performed due to the invasive nature of the test. Conventional pulmonary angiography lacks precision in diagnosing pulmonary embolism that is confined to subsegmental arteries [94]. For instance, one study reported a possibility of misdiagnosis in ~33% of subsegmental emboli and ~33% of solitary subsegmental emboli on pulmonary angiograms initially [95]. Procedure-related complications are also a concern. Among the 1111 patients that underwent angiography in PIOPED, complications of death occurred in five patients, renal dysfunction in thirteen, respiratory distress in four, and hematoma in two patients [77]. However, it is still a justifiable diagnostic technique in the proper clinical context [96].

### 8.6. Echocardiography

The major utility of an echocardiogram during acute PE is its ability to assess for right ventricular strain and elevated risk for poorer outcomes [97]. Both pulmonary artery enlargement and cardiomegaly do not demonstrate sensitivity or specificity in detecting the echocardiographic manifestation of right ventricular hypokinesis, which is a significant predictor of death in cases of acute pulmonary embolism [98]. Transthoracic echocardiography (TTE) is commonly used to investigate right ventricular (RV) pressure overload in individuals suspected of having acute PE [99]. McConnell’s sign, a specific echocardiographic pattern characterized by localized right ventricular failure with the apex being unaffected, can occur in some PEs (77% sensitivity; 94% specificity), but it is not a specific indicator of pulmonary embolism [100,101,102]. Other findings such as right ventricle/left ventricle size ratio, septal motion abnormality, tricuspid regurgitation, 60/60 sign, hypokinesis of right ventricle, pulmonary hypertension, right ventricular end-diastolic diameter, tricuspid annular plane systolic excursion (TAPSE), and right ventricular systolic pressure can also be evaluated on an echocardiogram [77,103,104,105].

A meta-analysis of 511 patients with pulmonary embolism and transthoracic echocardiography showed that 71% of patients with PE had no significant abnormalities on TTE [106]. In patients that had TTE findings, ~27% had RV enlargement, ~27% had RV free wall hypokinesis while ~20% had the McConnell sign, 18% had interventricular septal flattening, and 13% had a 60/60 sign [107]. This study also reported that the simultaneous presence of hypokinetic right ventricle along with the 60/60 sign and the McConnell sign to be the most reliable indicator for RV strain [106].

The pulmonary embolism severity index (PESI)-Echo score (PESI + PASP-TAPSE = PESI-Echo) has been reported as an innovative and novel measure to evaluate the risk of mortality in individuals who have acute pulmonary embolism [108]. A multicentric prospective study among 684 patients in 75 academic centers in Argentina showed a PESI-Echo score greater than or equal to 128 as the optimal cutoff point to predict mortality while in the hospital (sensitivity 82%, specificity 69%) [108].

Although very rare, if there is a thrombus within the proximal pulmonary arteries and in the right atrium/right ventricle, this may also be visualized on an echocardiogram. A total of 1.8% of patients in the meta-analysis mentioned earlier had right heart thrombus. The presence of right heart thrombi in patients is mostly associated with the hemodynamic effects of pulmonary embolism rather than the specific characteristics of thrombi. Nevertheless, individuals with right heart thrombi and pulmonary embolism leading to right ventricular dysfunction appear to have a worse outcome compared to controls matched based on propensity scores [109].

### 8.7. Role of Point-of-Care Ultrasound for Diagnosis of PE in the Modern Era

Point-of-care ultrasound (POCUS) is a fast, safe, effective, and valuable tool that is available at the bedside which can aid in diagnosis if integrated with traditional clinical examination. In acute settings, the POCUS evaluation helps to assess evidence of right heart strain, which is particularly useful if the patient is hemodynamically unstable to travel for imaging or has renal impairment or other contraindications to obtain CTPA urgently. One of the major limitations is operator dependency. Similarly, POCUS cannot distinguish other causes of right heart strain, such as RV infarction, and cannot be used to exclude the diagnosis of PE, as a lack of RV strain does not necessarily rule out PE [110]. More recent studies are also exploring triple point-of-care US (heart, lung, and venous compression ultrasound) for a real-time assessment, which has promising potential but is not yet a formally recommended alternative diagnostic approach [111].

### 8.8. Compression Ultrasonography

Thrombi are often formed in the lower extremities and embolize to the lungs. As a result, venous compression ultrasound (CUS) is often performed in patients suspected of having DVT/PE. A combination of lower-extremity ultrasound and echocardiography may also offer increased specificity (if positive) or negative predictive value (if negative) in patients who cannot have a CTPA for some reason [112]. However, CUS has low sensitivity (sensitivity, 41%; 95% CI, 36–46%) and, therefore, cannot be used to rule out PE [113]. A retrospective study of 168 patients with acute PE showed that 46.4% of patients had a negative lower-extremity venous compression ultrasound [114]. Negative CUS was more often seen in patients with no history of DVT, low D-dimer levels, PE on V/P-SPECT rather than CT, and peripheral PEs [115].

## 9. Conclusions

Pulmonary embolism remains a major contributor to cardiovascular mortality despite many advances in diagnostic technologies over the last few decades. Clinical judgment and validated risk assessment tools should be used to guide diagnosis to reduce unnecessary testing. The presence of various clinical and laboratory features in patients can provide hints for diagnosis and indicate characteristics that can reduce the chances of erroneously ruling out the diagnosis of PE. Laboratory testing and imaging may be indicated in patients with intermediate and high risk for PE. Appropriate risk stratification is crucial for both the diagnosis and management of these patients.

## Figures and Tables

**Table 1:** Common clinical manifestations of pulmonary embolism.

Clinical Features	Physical Examination Findings
Dyspnea	Tachycardia
Pleuritic chest pain	Tachypnea
Cough	Hypotension/Shock
Hemoptysis	Hypoxemia
Syncope	Orthostatic dysfunction
	Cardiac arrythmias
	JVD: Jugular venous distention
	Loud pulmonic heart sound
	Right ventricular parasternal lift

**Table 2:** Clinical scoring systems.

	Wells Score [22]		Revised Geneva Score [23]	
	Original	Simplified	Original	Simplified
Previous DVT or PE	1.5	1	3	1
Heart rate				
75–94/min			3	1
>=95/min			5	2
>100/min	1.5	1		
Surgery/fracture/immobilization within 4 weeks (1 month)	1.5	1	2	1
Hemoptysis	1	1	2	1
Cancer (active)	1	1	2	1
Clinical signs of DVT	3	1		
One-sided limb pain			3	1
Pain on calf palpation (Homan’s positive) and unilateral edema			4	1
Alternative diagnosis less likely than PE	3	1		
Age > 65 years			1	1
**PE unlikely**	**<=4**	**<=1**	**<=5**	**<=2**
**PE likely**	**>4**	**>1**	**>5**	**>2**

DVT: Deep vein thrombosis; PE: Pulmonary embolism.

**Table 3:** A: Sensitivity, Specificity, and Negative Predictive Value of commercially available D-dimer assays, Pro-BNP, and Troponin for Detection of Venous Thromboembolism. B: Utility of BNP or NT-Pro-BNP in Prognostication of Pulmonary Embolism. Adapted from two metanalyses where BNP/NT-Pro-BNP was utilized to assess short-term mortality, PE-related mortality, and serious adverse events. C: Associations of Different Troponin Assays with Outcomes. Pooled odds ratio was utilized to assess mortality in acute PE using various troponin assays.

**A**					
**Biomarker**	**Sensitivity** **VTE (%)**	**Specificity** **VTE (%)**	**NPV** **VTE (%)**	**Sensitivity DVT (%)**	**Sensitivity PE (%)**
* D-Dimer					
Enzyme-linked immunofluorescence assay (ELFA)	96–97	57	99	96	97
Microplate enzyme-linked immunosorbent assay (ELISA)	95	45	97	94	95
Latex quantitative assay	95	48–61	99	93	95
Whole-blood D-dimer assay	75–87	69–83	89	83	87
Latex qualitative assay	75	99	99	69	75
Pro-BNP	85	80			
Troponin-I	65	42			
**B**					
**Outcomes**	**Sensitivity Study 1/** **Study 2** **(%)**	**Specificity Study 1/** **Study 2** **(%)**	**PPV** **Study 1/** **Study 2** **(%)**	**NPV Study 1/** **Study 2** **(%)**	**OR** **Study 1/** **Study 2**
Short-term death	93/96	48/42	14/13	99/99	6.57/7.7
Death resulting from PE	92/97	52/42	13/12	99/97	6.10/6.4
Serious adverse events	89/100	48/36	33/26	94/100	7.47/15.6
**C**					
**Outcomes**	**All Troponins**	**Conventional** **Troponin-I**	**Conventional** **Troponin-T**	**High-Sensitivity** **Troponin**	
	**OR (95% CI)**	**OR (95% CI)**	**OR (95% CI)**	**OR (95% CI)**	
Overall mortality	4.3 (3.3–5.7)	2.8 (2.0–4.0)	7.9 (4.5–13.6)	3.7 (1.2–11.6)	
Short-term mortality	5.2 (3.3–8.4)				
PE-related mortality	9.4 (4.1–21.5)				
Adverse outcomes	7.0 (2.4–20.4)				
90-day mortality	4.8 (2.8–8.2)				
Mortality in low-risk PE subgroup				6.9 (1.3–35.8)	

A: Adapted from (reference/s): [34,36,39,40]. BNP: brain natriuretic peptide; NPV: negative predictive value. B: Adapted from (reference/s): [39,41,42]. NT-Pro-BNP: N-terminal pro–B-type natriuretic peptide; PE: pulmonary embolism; PPV: positive predictive value; NPV: negative predictive value, OR: odds ratio. C: Adapted from (reference/s): [43,44,45,46,47,48]. OR: odds ratio; 95% CI: 95% confidence interval; PE: pulmonary embolism. * D-dimer measurement, preferably high-sensitivity assay, with age-adjusted cutoff (age × 10 µg/L in patient with age > 50 yrs) is recommended in outpatient/emergency-room patients with low or intermediate clinical probability/PE unlikely. D-dimer measurement is not recommended in patient with high clinical probability as a negative test does not rule out PE.

**Table 4:** Comparison of major guidelines on risk stratification and diagnosis of pulmonary embolism.

Guidelines	Categories	Risk Stratification	Diagnosis
ESC 2019 [7]	Low Risk	0 to 3 on revised Geneva or 0 to 1 on modified simplified Geneva score	History + risk assessment PERC rule
	Intermediate Risk	4 to 10 on revised Geneva or 2 to 4 on modified simplified Geneva score	History + risk assessment Age adjusted D-dimer
	High Risk	11 to 25 on revised Geneva or >5 on modified simplified Geneva score	CTPA vs. V/Q SPECT
ACC/AHA 2011 [44]	Non-Massive	Normotensive, normal Biomarkers, and PE unlikely in sPESI (or PESI)	
	Submassive	PESI class III-IV or sPESI ≥ 1, echo or CT evidence of RV strain, positive troponin, or elevated BNP or NT-Pro-BNP	
	Massive	Hypotension (systolic blood pressure < 90 mm Hg for ≥15 min, drop in systolic blood pressure of ≥40 mm Hg or vasopressor), or thrombus in transit, or syncope, or cardiac arrest	

ESC: European Society of Cardiology; ACC: American College of Cardiology; AHA: American Heart Association; CT: computerized tomography; CTPA: CT pulmonary angiogram; V/Q: ventilation- perfusion; SPECT: single-photon emission CT; PE: pulmonary embolism; PERC: PE Rule-out Criteria; PESI: PE severity index; NT-Pro-BNP: N-terminal Pro–B-type natriuretic peptide.
